# An insight into the roles of ubiquitin-specific proteases in plants: development and growth, morphogenesis, and stress response

**DOI:** 10.3389/fpls.2024.1396634

**Published:** 2024-06-27

**Authors:** Xiuwen Wang, Xuan Liu, Kaixuan Song, Liang Du

**Affiliations:** ^1^ State Key Laboratory of Tree Genetics and Breeding, College of Biological Sciences and Technology, Beijing Forestry University, Beijing, China; ^2^ Key Laboratory of Genetics and Breeding in Forest Trees and Ornamental Plants, Ministry of Education, College of Biological Sciences and Technology, Beijing Forestry University, Beijing, China

**Keywords:** ubiquitin-specific protease, deubiquitination, plant development and growth, morphogenesis, plant stress response

## Abstract

Ubiquitination is a highly conserved and dynamic post-translational modification in which protein substrates are modified by ubiquitin to influence their activity, localization, or stability. Deubiquitination enzymes (DUBs) counter ubiquitin signaling by removing ubiquitin from the substrates. Ubiquitin-specific proteases (UBPs), the largest subfamily of DUBs, are conserved in plants, serving diverse functions across various cellular processes, although members within the same group often exhibit functional redundancy. Here, we briefly review recent advances in understanding the biological roles of UBPs, particularly the molecular mechanism by which UBPs regulate plant development and growth, morphogenesis, and stress response, which sheds light on the mechanistic roles of deubiquitination in plants.

## 1 Introduction

Protein post-translational modifications are chemical reactions that modify proteins after their synthesis. These modifications, such as phosphorylation, acetylation, methylation, glycosylation, and ubiquitination, greatly enrich the functional diversity of proteins and markedly affect their structure, stability, activity, and localization (Pejaver et al., 2014; Lee et al., 2023). Ubiquitination influences protein fate and plays a role in regulating various biological processes, including cell-cycle regulation, DNA damage repair, endocytosis, and stress and immune responses (Lyzenga and Stone, 2012; Dubeaux and Vert, 2017; Zou and Lin, 2021; Gao et al., 2022; Koo et al., 2023). Ubiquitination involves the covalent attachment of ubiquitin molecules, typically to lysine residues of proteins, catalyzed by the sequential activity of three types of enzymes, comprising ubiquitin-activating enzyme E1, ubiquitin-conjugating enzyme E2, and ubiquitin-ligating enzyme E3. In some cases, these enzymes link ubiquitin proteins to target proteins via a cascade reaction, forming ubiquitin chains (Komander and Rape, 2012). In general, the fate of ubiquitinated proteins is determined by the ubiquitin chain linkage type and the number of ubiquitin units bound (Singh et al., 2016). Protein ubiquitination can be reversed by the activity of deubiquitinases or deubiquitination enzymes (DUBs) (Komander et al., 2009; Skelly, 2022). Together, deubiquitination acts antagonistically with ubiquitination to form a dynamic network of protein ubiquitination modification within cells, ensuring the normal functioning of cellular processes (Sahtoe and Sixma, 2015; Cai et al., 2018).

**Figure 1:**
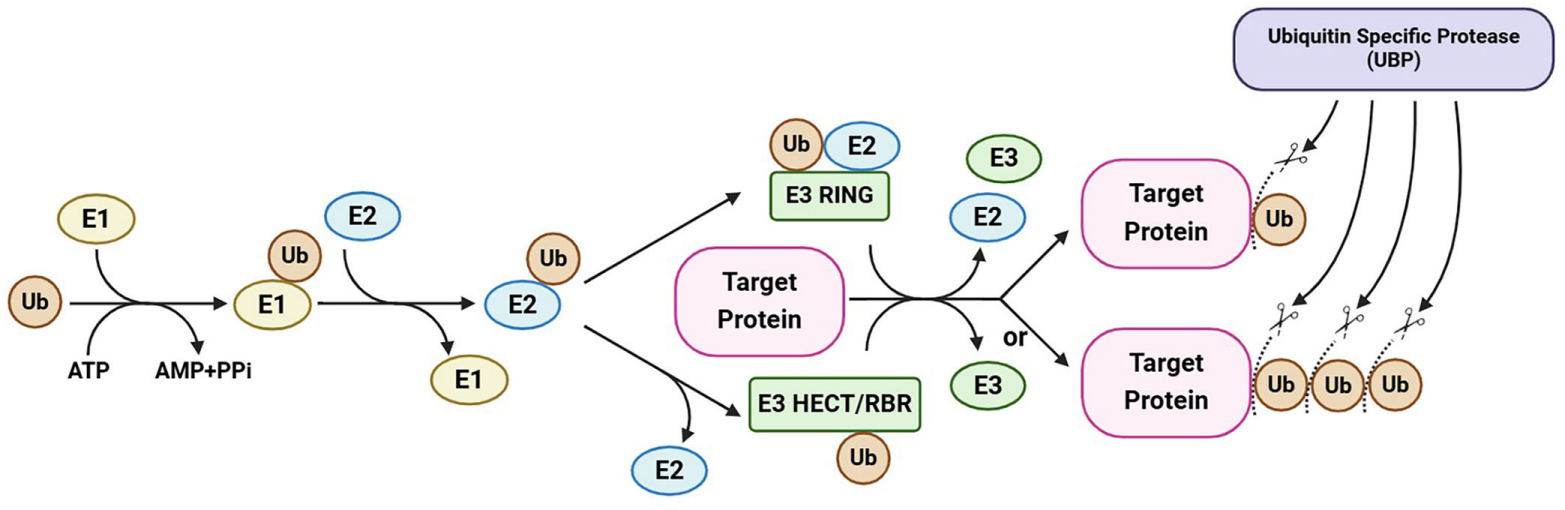
A simplified illustration of protein ubiquitination and deubiquitination. Ubiquitination and deubiquitination regulate the fate and activity of target proteins through the addition and removal of ubiquitin (chain). Ubiquitination is catalyzed by E1, E2, and E3 enzymes in sequential steps and can be reversed by ubiquitin-specific proteases (the largest subfamily of deubiquitinases), which cleave ubiquitin chains. This maintains a balance between protein ubiquitination and deubiquitination, regulating the stability and/or activity of a target protein. This figure was created on the BioRender website (https://app.biorender.com/).

Ubiquitin-specific proteases (UBPs), the largest subfamily of DUBs, are hydrolases unique to plants (Isono and Nagel, 2014). The UBP members participate in the generation of free ubiquitin monomers from ubiquitin precursors, the recycling of ubiquitin during the decomposition of ubiquitin–protein conjugates, and the removal of ubiquitin from target proteins, thereby potentially preventing their degradation (**Figure 1**) (Amerik and Hochstrasser, 2004; Nijman et al., 2005; Clague et al., 2012). Increasing numbers of recent studies have enhanced our understanding of the molecular mechanisms underlying the cellular and physiological functions of UBPs in plants. Here, we outline the molecular characterization and identification of plant UBPs and summarize the roles of UBPs in plant development and growth, morphogenesis, and stress response (**Table 1**). This review will contribute to an improved systematic understanding of the molecular functions of plant deubiquitinases and promote the use of UBP-related gene resources in crop improvement.

**Table 1:** A summary of plant UBP traits and functions.

Sub-family	Name	Subcellular Localization	Non-UBP domains	Function Development	Morphogenesis	Stress response	Reference
1	UBP1	nucleus^*^	Zf-UBP	–	–	canavanine resistance	(Yan et al., 2000)
	UBP2	nucleus^*^	Zf-UBP	–	–	canavanine resistance, cell death and disease resistance	(Yan et al., 2000; Jiang et al., 2022a)
2	UBP3	nucleus	–	pollen development and transmission	–	–	–
	UBP4	nucleus	–	pollen development and transmission	–	–	(Doelling et al., 2007)
3	UBP5	nucleus^*^	DUSP	H2Aub1 deubiquitinase	–	–	(Zheng et al., 2023)
	UBP8	nucleus^*^	–	–	–	–	–
	UBP9	nucleus^*^	DUSP	–	–	–	–
	UBP10	nucleus^*^	DUSP	–	–	–	–
	UBP11	nucleus^*^	DUSP	–	–	–	–
4	UBP6	cytosol^*^	UBL	–	–	canavanine resistance	(Moon et al., 2005)
	UBP7	nucleus^*^	UBL	–	–	–	–
5	UBP12	cytoplasm and nucleus	MATH	root meristem / leaf development., flowering, circadian rhythms, seed development, H2Aubl deubiquitinase	Photomorphogenesis	JA response, BR / ABA signaling, plant immunity, shade avoidance response, drought resistance, DNA damage response, UV tolerance	(Ewan et al., 2011; Cui et al., 2013; Derkacheva et al., 2016; Jeong et al., 2017; An et al., 2018; Al Khateeb et al., 2019; Vanhaeren et al., 2020; Lim et al., 2021a; Zhou et al., 2021; Lan et al., 2022; Lindback et al., 2022; Liu et al., 2022; Luo et al., 2022; Hu et al., 2023)
	UBP13						
6	UBP14	nucleus	Zf-UBP. UBA	leaf size and endoreduplication, embryo development	–	root hair development (phosphate deficiency), auxin response	(Doelling et al., 2001; Li et al., 2010; Xu et al., 2016; Majumdar et al., 2020; Guo et al., 2024)
7	UBP15	cytoplasm and nucleus	Zf-MYND	cell proliferation, flowering, organ size control	–	Cd and salt stress response	(Du et al., 2014; Kong et al., 2019; Shi et al., 2019)
	UBP16	nucleus	Zf-MYND	floral development	–	salt/cadmium tolerance, Cd stress response	(Zhou et al., 2012; Zhao et al., 2013; Kong et al., 2019; Pamei and Makandar, 2022)
	UBP17	nucleus^*^	Zf-MYND	–	–	–	–
	UBP18	nucleus^*^	Zf-MYND	–	–	–	–
	UBP19	nucleus^*^	Zf-MYND	embryo development	–	Cd and salt stress response	(Liu et al., 2008; Kong et al., 2019)
8	UBP20	nucleus^*^	–	–	–	–	–
	UBP21	nucleus^*^	–	–	–	–	–
9	UBP22	nucleus	Zf-UBP	H2Bub1 deubiquitinase	Photomorphogenesis	–	(Nassrallah et al., 2018)
10	UBP23	nucleus^*^	–	–	–	–	–
11	UBP24	nucleus^*^	–	–	–	ABA signaling, drought tolerance	(Zhao et al., 2016)
12	UBP25	nucleus^*^	–	–	–	–	–
13	UBP26	nucleus	DUSP. UBL	floral development, seed development, H2Bub1 deubiquitination	–	–	(Luo et al., 2008; Schmitz et al., 2009)
14	UBP27	mitochondria	–	–	mitochondria morphogenesis	–	(Pan et al., 2014)

In the subcellular localization column, those with ^*^ are predicted from the CELLO website: http://cello.life.nctu.edu.tw/ , and those without ^*^ are reported in the previous research.

## 2 Ubiquitin and ubiquitination

Ubiquitin is a highly conserved small globular protein consisting of 76 amino acids that is synthesized across all eukaryotes. The multifunctionality of ubiquitin in regulating biological processes is attributed to its ability to attach to proteins as a monomer (monoubiquitination) or to multiple lysine residues on substrates (multiubiquitination) or to act as a polymer by sequentially adding other ubiquitin molecules to an existing ubiquitin chain (polyubiquitination) from a lysine residue (Mevissen and Komander, 2017; Romero-Barrios and Vert, 2018). Polyubiquitination can be subclassified further into linear polyubiquitination and branching polyubiquitination depending on how the ubiquitin moieties are connected (Sadowski et al., 2012; Mevissen and Komander, 2017). The type of ubiquitination determines the biological outcomes. Monoubiquitination and multiubiquitination mainly affect protein trafficking and protein–protein interactions (Liu et al., 2021), whereas polyubiquitination induces more complex cellular events, such as proteasomal degradation, signaling activation, and endocytosis (Li and Ye, 2008; Kim et al., 2013). In plants, monoubiquitin conjugation affects plant immunity by regulating susceptibility to bacterial and fungal pathogens (Ma et al., 2020). In addition, ubiquitination can be achieved by linking ubiquitin to cysteine, serine, threonine, and the N-terminal amino acid of the substrate protein (Cadwell and Coscoy, 2005; Wang et al., 2007; Williams et al., 2007; McDowell and Philpott, 2013). These types of ubiquitination modifications can alter the biological activities of substrate proteins (Ohtake et al., 2015; Mevissen and Komander, 2017). However, the functional relevance of atypical ubiquitinated target sites remains unclear and requires further investigation.

Ubiquitination is a highly conserved post-translational modification, playing versatile roles in cell cycle/division, differentiation, endocytosis, vesicle trafficking, transcriptional regulation, and immune and stress responses, in both plants and animals (Dubeaux and Vert, 2017; Gilberto and Peter, 2017; Zhou and Zeng, 2017; Song and Luo, 2019; Sharma et al., 2021). Hence, ubiquitination dysregulation can have detrimental effects on cells. The ubiquitination regulatory pathway involves a three-step enzymatic cascade comprising ubiquitin-activating enzyme E1, ubiquitin conjugating-enzyme E2, and ubiquitin ligase E3 (Hershko and Ciechanover, 1998; Pickart, 2001; Komander and Rape, 2012). This cascade results in ubiquitin transfer, via its C-terminal glycine, onto the ε-amino group of a lysine residue on the substrate (Komander and Rape, 2012; Zheng and Shabek, 2017). Cells exhibit strong disparity in the diversity of each of these three enzyme groups. For example, the human genome has more than 600 E3 enzymes, but only 41 E2 and eight E1 enzymes (Liu et al., 2019; Huang et al., 2024). This imbalance is more pronounced in plants. For example, the *Arabidopsis* genome, which is less than 1/20th the size of the human genome, encodes more than 1,400 E3 members, 37 E2, and two E1 enzymes (Vierstra, 2012; Al-Saharin et al., 2022; Skelly, 2022), suggesting greater complexity in the roles of plant E3 enzymes in conferring substrate specificity (Serrano et al., 2018; Block et al., 2023).

## 3 Deubiquitination and plant UBP

**Figure 2:**
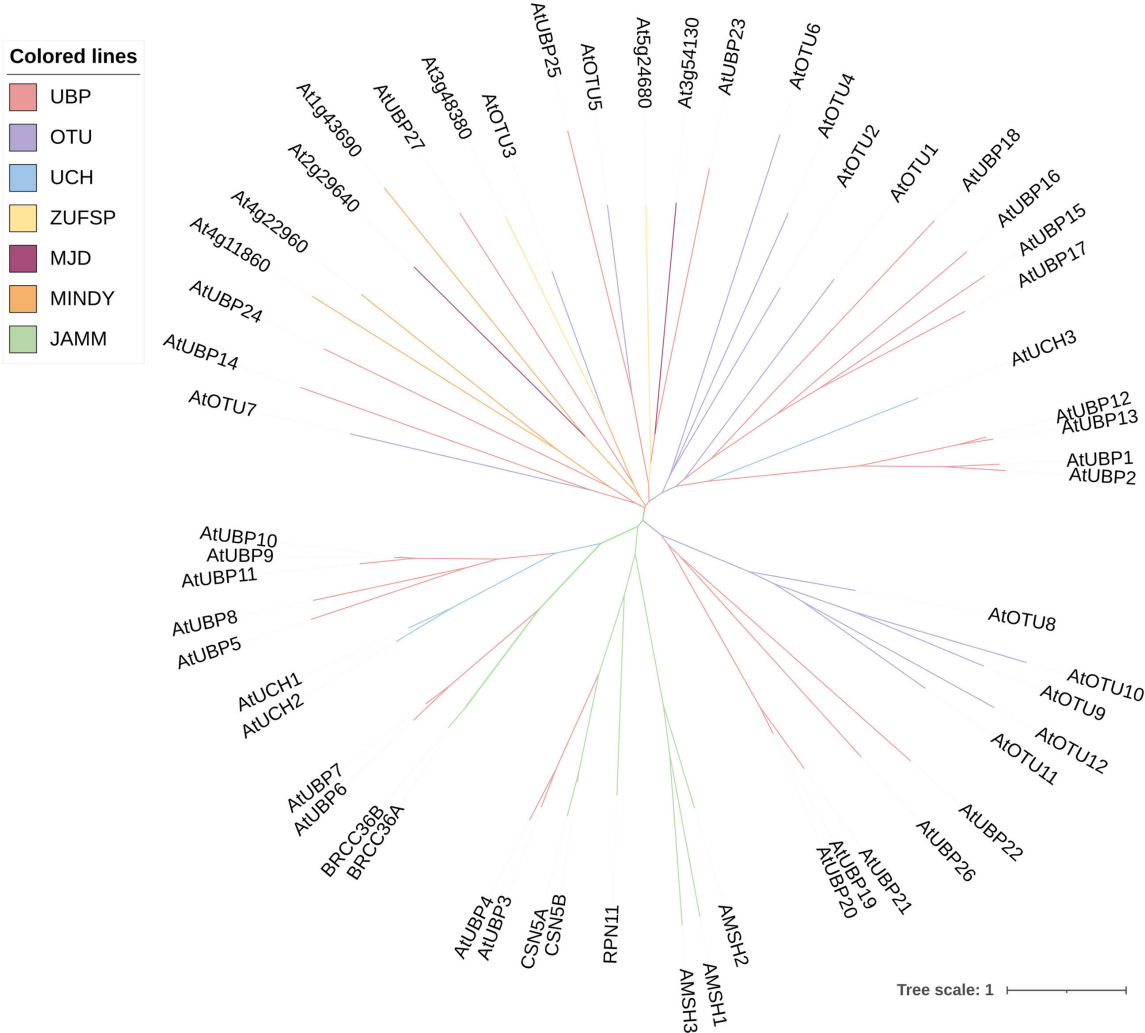
Phylogenetic relationships among DUB family proteins in *Arabidopsis*. The phylogenetic tree was constructed using MEGA11 software by the neighbor-joining method with 1,000 bootstrap replicates. The tree was modified on the iTOL website (https://itol.embl.de/). Different colors of branches indicate distinct DUB subfamilies.

Deubiquitination is the process by which DUBs remove ubiquitin molecules from proteins that have been modified by ubiquitination. This is the reverse process of protein modification by ubiquitination, which is crucial for maintaining the stability, function, and balance of intracellular protein abundance (Isono and Nagel, 2014; Luo et al., 2023). In addition to removing the ubiquitin chain from the substrate protein, DUBs are involved in processing ubiquitin precursors and editing the ubiquitin chain (Amerik and Hochstrasser, 2004; Nijman et al., 2005). It was previously believed that *Arabidopsis* DUBs involved just five subfamilies (Yan et al., 2000). However, according to a recent study, two more subfamilies have been added: motif interacting with ubiquitin-containing novel DUB family (MINDY) and zinc finger with UFM1-specific peptidase (ZUFSP) domain proteases (Luo et al., 2023). Therefore, plant DUBs can be classified into at least seven subfamilies, comprising ubiquitin-specific proteases (UBPs or USPs), ubiquitin C-terminal hydrolases (UCHs), ovarian tumor proteases (OTUs), Machado-Joseph domain proteases (MJD), MINDY, ZUFSP, and JAB1/MPN/MOV34 (JAMM) proteases (Komander et al., 2009; Clague et al., 2019; Luo et al., 2023). The first six subfamilies are all cysteine proteases, whereas the final subfamily belongs to the zinc metalloproteinase family (Clague et al., 2013; Skelly, 2022; Luo et al., 2023). In stark contrast to the vast number of E3 enzymes, the presence of DUBs in cells is comparatively limited. For example, the *Arabidopsis* genome encodes fewer than 70 DUBs according to recent research (**Figure 2**) (Trulsson et al., 2022; Luo et al., 2023). Owing to the functional antagonism between E3 and DUB molecules, the scarce DUBs might exhibit greater complexity and diversity in their operational roles.

**Figure 3:**
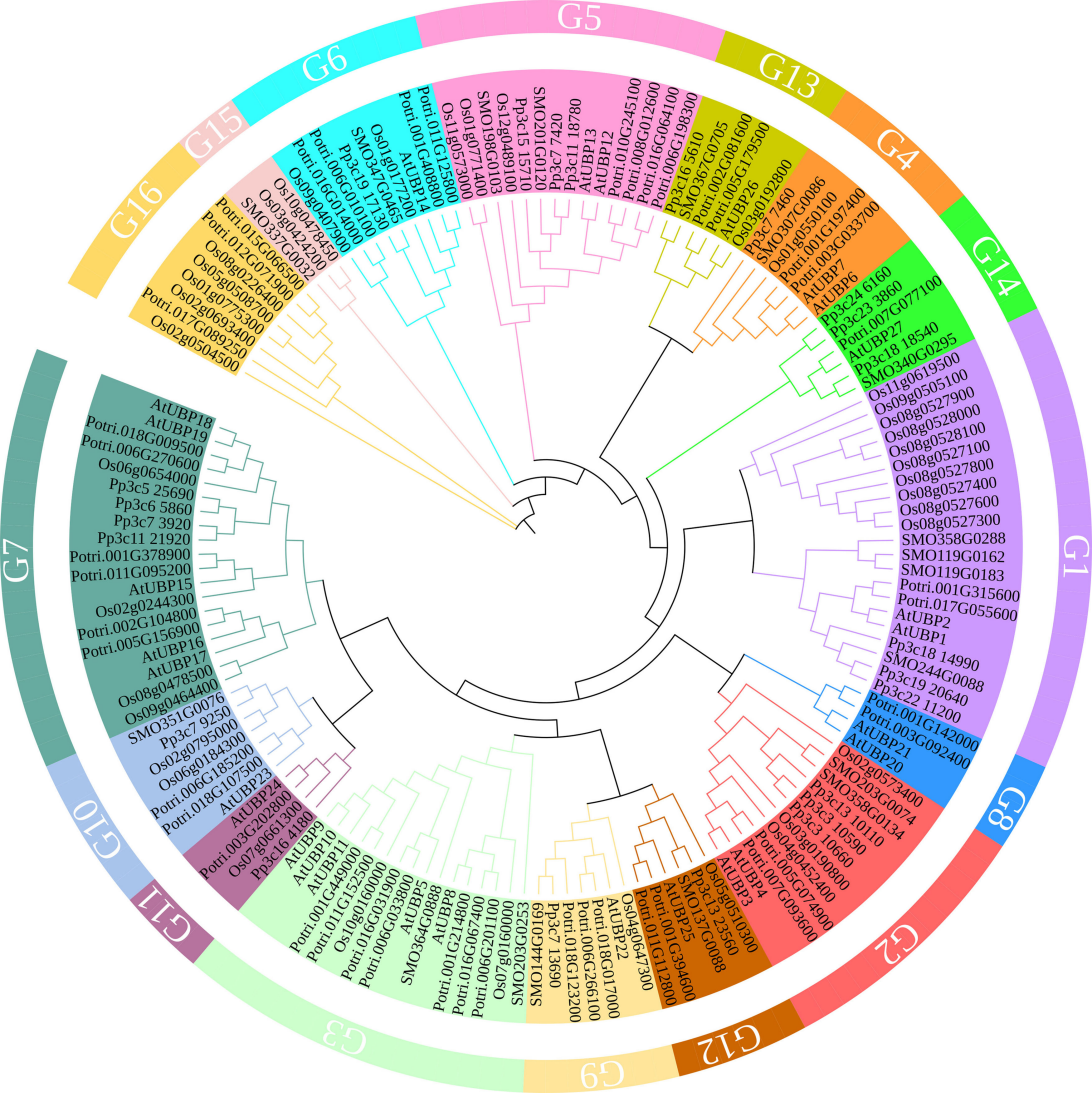
A phylogenetic tree of UBP family proteins from *Arabidopsis thaliana*, *Oryza sativa*, *Populus trichocarpa*, *Selaginella moellendorffii*, and *Physcomitrium patens*. The tree was constructed with MEGA11 software using the neighbor-joining method with 1,000 bootstrap replicates. The tree was modified on the iTOL website (https://itol.embl.de/). Protein sequences were obtained from published papers (Wang et al., 2018; Wu et al., 2019b; Zheng and Du, 2021) and downloaded from the PLAZA database (https://bioinformatics.psb.ugent.be/plaza/versions/plaza_v5_dicots/).

The UBPs are the most abundant DUB subfamily in plants. The model plant *Arabidopsis thaliana* has 27 UBP members, which constitute almost 40% of the DUB family (**Figure 2**). Plant UBPs can be divided into 14 subfamilies based on their sequence similarity and genomic organization, particularly their characteristic domains and motifs (Yan et al., 2000; Liu et al., 2008; Komander et al., 2009). The number of UBP members varies with the genome size of different plants, but given their highly conserved functions, such variation is limited (**Table 2**). For example, there are 44 UBPs in rice (*Oryza sativa*), 44 in *Populus trichocarpa*, 45 in maize (*Zea mays*), 21 in *Vernicia fordii*, and 48 in Moso bamboo (Wang et al., 2018; Wu et al., 2019a; Zheng and Du, 2021; Cao et al., 2022; Fu et al., 2024). Here, we constructed a phylogenetic tree for UBPs from five representative plant species: the dicotyledonous herb *A. thaliana*, the monocotyledonous *O. sativa*, the dicotyledonous woody tree *P. trichocarpa*, the lycophyte *Selaginella moellendorffii*, and the bryophyte *Physcomitrium patens*. Referring to the molecular characterization, plant UBP members can be divided into 14–16 groups, among which groups 1–14 correspond to the recognized groups in *A. thaliana* (Wu et al., 2019a; Xu et al., 2021; Zheng and Du, 2021). Most of these groups are present in all plants, such as groups 1–7 and 9–13; others are found only in certain plants, such as group 8, which is exclusive to *A. thaliana* and *P. trichocarpa* (**Figure 3**). Recent studies have shown that UBPs play crucial roles in plant development and growth, morphogenesis, and stress response (**Table 1**). UBP interaction partners are gradually being identified, significantly advancing our understanding of the molecular mechanisms of UBPs (**Table 3**). However, identification of the substrates of UBPs, a crucial aspect of understanding their function as deubiquitinating enzymes, remains a slow process, potentially representing a crucial challenge that requires addressing in the future.

**Table 2:** An overview of UBP gene families in different plants.

Species	Number of UBPs	References
*Arabidopsis thaliana*	27	Wilkinson (1997); Yan et al. (2000)
Moso bamboo	48	Wu et al. (2019a)
*Triticum aestivum*	97	Xu et al. (2021)
*Oryza sativa*	44	Wang et al. (2018)
*Brassica rapa*	41	Karamat et al. (2023)
*Populus trichocarpa*	44	Zheng and Du (2021)
*Vernicia fordii*	21	Cao et al. (2022)
*Vernicia montana*	26	Cao et al. (2022)
*Jatropha curcas*	24	Cao et al. (2022)
*Ricinus communis*	21	Cao et al. (2022)
*Manihot esculenta*	34	Cao et al. (2022)
*Hevea brasiliensis*	44	Cao et al. (2022)
*Zea mays*	45	Fu et al. (2024)
*Physcomitrium patens*	23	Wu et al. (2019b)
*Selaginella moellendorffii*	18	Wu et al. (2019b)
Three green algae	14	Wu et al. (2019b)

**Table 3:** A summary of *Arabidopsis* UBP interacting proteins.

UBPs	Interacting partner	Reference
UBP1	UBA1a, UBA2a	Lambermon et al. (2002)
UBP5	PWWP/H2A	Zheng et al. (2023)
UBP6	Calmodulin	Moon et al. (2005)
UBP12/13	RGFR1	An et al. (2018)
	MYC2	Jeong et al. (2017)
	UPL3	Lan et al. (2022)
	DA1/DAR1/DAR2	Vanhaeren et al. (2020)
	PIF7	Zhou et al. (2021)
	CRY2/COP1	Lindback et al. (2022)
	UVSSA/TFIIS	Al Khateeb et al. (2019)
	CPD/RAD51	Hu et al. (2023)
	BRI1	Luo et al. (2022)
	BES1	Park et al. (2022); Xiong et al. (2022)
	VPS23A	Liu et al. (2022)
	LHP1/PcG/H2A	Derkacheva et al. (2016)
	LHY/CCA1/TOC1	Cui et al. (2013)
	NPR3	Zhou et al. (2023)
	CaSnRK2.6	Lim et al. (2021b)
	GI	Lee et al. (2019)
	ATL31	Luo et al. (2022)
UBP14	UVI4	Xu et al. (2016)
	MAC3A/MAC3B	Guo et al. (2024)
	CDKB1;1/CYCA2;3	Xu et al. (2016); Jiang et al. (2022b)
UBP15	DA1	Du et al. (2014)
	LAS/CUC2/CUC3	Li et al. (2020)
UBP16	SHM1/SHM4	Zhou et al. (2012)
	HIPP27	Zhao et al. (2013)
UBP22	H2B	Nassrallah et al. (2018)
UBP24	ABI2	Zhao et al. (2016)
UBP26	FLC/H2B	Schmitz et al. (2009)
	PHE1	Luo et al. (2008)
UBP27	DRP3	Pan et al. (2014)

## 4 Structure of plant UBPs

**Figure 4:**
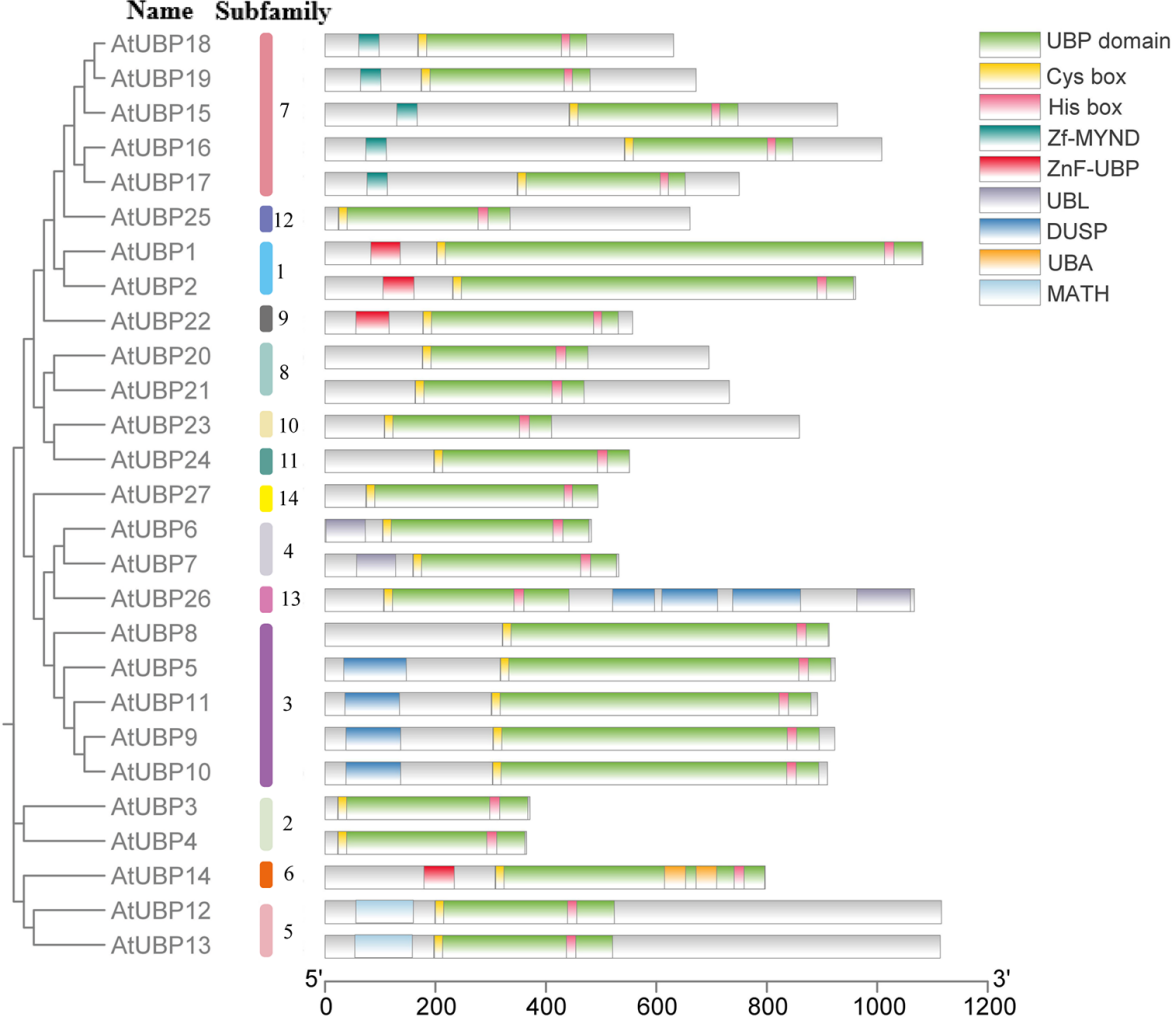
Conserved domains in *Arabidopsis thaliana* UBP proteins. Different colors represent different conserved domains. The SMART database (https://smart.embl.de/) and PROSITE database (https://prosite.expasy.org/) were used for this analysis.

In plants, ubiquitin affinity chromatography and anion-exchange high-performance liquid chromatography (HPLC) initially revealed that UBP-like DUBs can hydrolyze attached ubiquitin via a peptide or isopeptide bond (Sullivan et al., 1990). Subsequently, 27 UBP enzymes were identified from *Arabidopsis* with UBP-like DUB activity (Wilkinson, 1997). All UBP proteins characteristically possess a specific UBP domain with two short but well-conserved motifs, crucial components of two triads of catalytic sites termed cysteine (Cys) and histidine (His) boxes (Yan et al., 2000; Liu et al., 2008). Both Cys- and His-boxes are necessary for UBP deubiquitination, but the His-box is less conserved than the Cys-box, and the length and sequence of the His-box are variable (Yan et al., 2000; Liu et al., 2008). In *Arabidopsis*, all UBP members share common conserved amino acid or non-UBP protein motifs, but members of different subfamilies show variation in protein domains (**Figure 4**) (Yan et al., 2000; Liu et al., 2008). For example, the UBP15/16/17/18/19 group features a signature myeloid-type zinc finger domain, Nervy, and DEAF-1 (Zf-MYND) domain, which was previously identified as a protein–protein interaction domain in eukaryotes (Lutterbach et al., 1998). The UBP5/9/10/11 group involves tripod-like (DUSP) domains, which are considered to play a role in both protein–protein interaction and substrate recognition (de Jong et al., 2006). The UBP12/13 group contains a meprin and TRAF homology (MATH) domain, which contributes to self-association and interaction with receptors associated with UBP proteins via expanding domain termini to the oligomerization center in non-plant organisms (Park et al., 1999; Ye et al., 1999; Sunnerhagen et al., 2002). Some non-UBP domains are distributed in several UBP groups, such as the ZnF-UBP domain present in the UBP1/2 group, UBP14, and UBP22 and the ubiquitin-like (UBL) domain found in the UBP6/7 group and UBP26.

**Figure 5:**
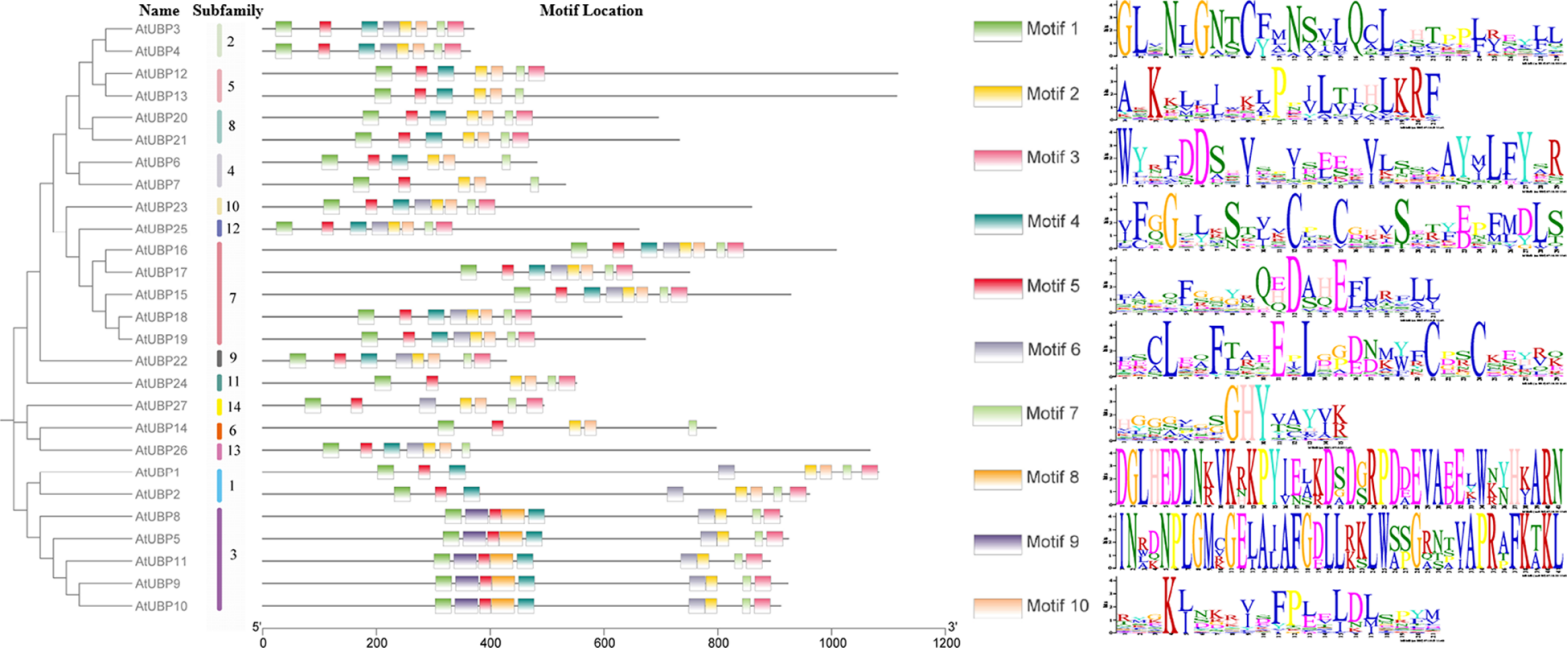
Motif distribution of *Arabidopsis* UBP proteins. Members of the same subfamily exhibit similar motif distribution characteristics. Different motifs are shown in differently colored rectangles, with the length of the black line representing protein size. AtUBP conserved motif analysis was performed on the MEME website (https://meme-suite.org/meme/).

We searched for the composition and diversification of 10 distinct motifs present in 27 AtUBP proteins using the Multiple Em for Motif Elicitation Suite (https://meme-suite.org/meme/) (**Figure 5**). The same group exhibits similar conserved motif distribution characteristics, suggesting that there may be functional redundancy. Furthermore, the type and order of motifs were identical in different subfamilies for motifs 1, 2, 3, 5, 7, and 8. Motifs 1, 2, 5, and 7 were present in all 27 UBP proteins, indicating their strong conservation in the AtUBP family. Motifs 8 and 9 were exclusively found in group 3 of the plant UBP family, whereas motif 10 was absent in group 3. These protein motifs or domains contribute to the identification of the unique functions of each group and, most importantly, provide evidence of diversity in their substrate specificity, localization, and physiological function (Amerik and Hochstrasser, 2004; Nijman et al., 2005; Clague et al., 2012; Isono and Nagel, 2014). However, the precise molecular mechanism by which these motifs or domains in plant UBP proteins function remains unclear.

## 5 UBPs play roles in plant development and growth

The development and growth of flowering plants are meticulously controlled throughout the life cycle to respond to internal signals and environmental stimuli. Mounting evidence indicates that UBPs are involved in almost all cellular events and thus play pivotal roles in plant development and growth.

### 5.1 Root meristem development: UBP12/13

ROOT MERISTEM GROWTH FACTOR 1 (RGF1) is an important peptide hormone that regulates root meristem development by influencing the expression of the two transcription factors *PLETHORA 1* and *2* (*PLT1/2*) (Mahonen et al., 2014). Activated RGF1 RECEPTOR 1 (RGFR1) is ubiquitinated and subsequently degraded by the 26S proteasome after ligand binding (Ou et al., 2016). UBP12 and UBP13 are regulators of the RGF1–RGFR1–PLT1/2 signaling pathway. By counteracting ligand-induced ubiquitination and degradation of RGFR1, UBP12 and UBP13 maintain the RGF1 sensitivity of root cells (An et al., 2018).

### 5.2 Axillary meristem initiation: UBP15

In seed plants, branching is achieved by the development of axillary meristems in the leaf axils, which has an important effect on plant architecture and crop yield (Grbic and Bleecker, 2000; Wang and Li, 2008). *CUP-SHAPED COTYLEDON 2* (*CUC2*) and *CUC3* encode members of the NAC transcription factor family and play essential roles in the regulation of axillary meristem initiation in *Arabidopsis*, thereby influencing the formation of plant branches (Hibara et al., 2006). UBP15, serving as a direct substrate for the peptidase DA1, suppresses the development of axillary meristems. In the CUC2/3–DA1–UBP15 pathway, the CUC2 and CUC3 proteins directly activate *DA1* expression, leading to greater degradation of UBP15 proteins (Li et al., 2020).

### 5.3 Leaf size and senescence: UBP12/13/14/15

The potential peptidases DA1 and DA1-related proteins can be activated through monoubiquitination mediated by two E3 ligases, BIG BROTHER (BB) and DA2, and subsequently disrupt the stability of various positive regulators in plant development and growth (Dong et al., 2017). UBP12 and UBP13 bind to, and remove ubiquitin from, these proteases, rendering them in an inactive state (Vanhaeren et al., 2020). Overexpression of *UBP12* or *UBP13* significantly reduces leaf size and cell area, leading to lower ploidy levels, whereas mutations that downregulate *UBP12* and *UBP13* result in smaller leaves with fewer and smaller cells, suggesting that UBP12 and UBP13 fine-tune plant leaf growth by restricting the peptidase activity of DA1 and DA1-related proteins (Vanhaeren et al., 2020). As a direct substrate of DA1, UBP15 redundantly exerts its effect with UBP16/17 on leaf size by regulating cell proliferation (Liu et al., 2008; Du et al., 2014; Dong et al., 2017). HECT-containing ubiquitin-protein ligase 3 (UPL3) plays critical roles in leaf senescence (Lan et al., 2022). In a recent study, UPL3 was shown to interact with UBP12 to form a regulatory hub for proteolysis-independent regulation and proteolysis-dependent degradation to control leaf senescence metabolically (Lan et al., 2022).

Leaf size is influenced by cell size, which is positively correlated with endopolyploidy (Gonzalez et al., 2010). During mitotic anaphase, the anaphase-promoting complex/cyclosome (APC/C) promotes ubiquitination and degradation of mitotic cyclins through the ubiquitin/26S proteasome, eventually affecting the endocycle (Zielke et al., 2008; Heyman and De Veylder, 2012). UBP14, encoded by *DA3*, serves as a negative regulator of endoreplication. Genetic analyses showed that UBP14 cooperates with the APC/C inhibitor UV-B-INSENSITIVE 4 (UVI4) and opposes the APC/C activator CELL CYCLE SWITCH 52 A1 (CCS52A1), whereas biochemical analyses showed that UBP14 physically interacted with UVI4 but not CCS52A1 (Xu et al., 2016). In addition, UBP14 influences the stability of two downstream components of APC/C: cyclin-dependent kinase B1;1 (CDKB1;1) and cyclin A2;3 (CYCA2;3) (Xu et al., 2016), which form a functional complex to suppress endocycle onset (Boudolf et al., 2009). Through genetic screening, significant progress has been made in clarifying the mechanism by which UBP14 regulates endoreplication, cell growth, and leaf size (Jiang et al., 2022b; Guo et al., 2024). CYCLIN-DEPENDENT KINASE G2 (CDKG2)/SUPPRESSOR OF *da3–1* 6 (SUD6) and the E3 ligases MOS4-ASSOCIATED COMPLEX 3A (MAC3A) and MAC3B are suppressors of the *ubp14* mutation (*da3–1*). CDKG2 physically interacts with CDKB1;1 acting downstream of UBP14-CDKB1;1 to control the ploidy level and leaf size (Boudolf et al., 2009; Jiang et al., 2022b), whereas MAC3A and MAC3B physically interact with and ubiquitinate UBP14 to modulate its stability (Guo et al., 2024).

### 5.4 Plant reproduction and growth

Flowering plants constitute approximately 90% of all known plant species, with the majority of them reproducing sexually (Amasino et al., 2017; Sauquet et al., 2017; Cronk, 2022). Plant sexual reproduction is a complex multistep process that involves cell fate specification and cell division, necessitating precise coordination of gene expression to respond to various signals (Golicz et al., 2018).

#### 5.4.1 Floral development: UBP3/4/12/13/15/26, SiUBP16

Pollen tube guidance by pistil tissue is essential for the delivery of non-motile sperm cells to female gametes during sexual reproduction in flowering plants (Higashiyama and Takeuchi, 2015). A series of regulatory processes governs pollen tube growth and development (Higashiyama and Takeuchi, 2015; Cameron and Geitmann, 2018). Two of the smallest UBPs, UBP3 and UBP4, function redundantly as regulators of pollen tube growth. Mutations in UBP3 and UBP4, taken individually, show no obvious phenotypic defects, whereas pollen of the *ubp3 ubp4* double mutant exhibits cell-cycle arrest at meiosis II of male gametogenesis together with defective vacuole and endomembrane organization (Doelling et al., 2007). In addition, abnormal pollen germination and transmission contribute to the infertility of *ubp3 ubp4* plants (Doelling et al., 2007).

The MADS-box transcription factor FLOWERING LOCUS C (FLC), which plays a role in the floral transition and acts as a negative regulator of flowering, represses the expression of numerous genes associated with flowering (Michaels and Amasino, 1999; Sheldon et al., 1999). Loss-of-function of *UBP26* causes accumulation of H2B monoubiquitination, together with a reduction in H3K36 trimethylation and an increase in H3K27 trimethylation at the *FLC* locus, leading to a decrease in the *FLC* mRNA level (Sridhar et al., 2007; Schmitz et al., 2009). Activation of FLC by UBP26 aligns with the model whereby deubiquitination is crucial for the accumulation of H3K36 trimethylation and maintaining the appropriate level of transcriptional activation (Sridhar et al., 2007; Schmitz et al., 2009).

A biological clock refers to the inherent rhythmicity that enables plants to respond to complex environmental changes. Through feedback loops between core components, plants integrate environmental cues and coordinate photoperiodic flowering, which is regulated by a CONSTANS (CO)-dependent signaling and circadian rhythm of clock genes, including *LATE ELONGATED HYPOCOTYL* (*LHY*), *CIRCADIAN CLOCK ASSOCIATED 1* (*CCA1*), and *TIMING OF CAB EXPRESSION 1* (*TOC1*) (Alabadi et al., 2001; Gendron et al., 2012; Huang et al., 2012). *UBP12* and *UBP13* are controlled by circadian rhythms, and double mutants of these genes exhibit pleiotropic phenotypes, including early flowering and a shortened circadian rhythm (Cui et al., 2013). In *ubp12 ubp13* mutants, *CO* transcription is elevated during the daytime earlier than in wild-type plants, leading to increased expression of *FLOWERING LOCUS T* (*FT*). UBP12 and UBP13 regulate the expression of clock genes, including the timing of *LHY*, *CCA1*, and *TOC1* expression (Cui et al., 2013). Interestingly, in a study of a polycomb group (PcG) function, the regulatory effect of UBP12/13 on flowering genes was similarly observed. PcG proteins are a diverse superfamily forming three major types of Polycomb repressive complexes (PRCs), namely, PRC1, PRC2, and polycomb repressive deubiquitinase. These proteins epigenetically inhibit the transcription of developmental genes by modifying and/or remodeling chromatin, thereby regulating gene expression (Chittock et al., 2017). UBP12 and UBP13 interact with the PcG-related protein LIKE HETEROCHROMATIN PROTEIN 1 (LHP1) and together regulate the degree of H3K27me3 modification, resulting in the silencing of PcG target genes (Derkacheva et al., 2016). Similar to the *lhp1* mutant, the *ubp12* mutant flowers earlier because of upregulation of *FT*, and the PcG target genes *FLC*, *MADS AFFECTING FLOWERING 4* (*MAF4*), and *MAF5* are upregulated in the *ubp12* and *ubp13* mutants (Derkacheva et al., 2016). In addition, insights into the molecular mechanism by which UBP12/13 contribute to the regulation of circadian rhythms have been reported. ZEITLUPE (ZTL) is a photoreceptor with E3 ubiquitin ligase activity that relays the end-of-day lighting conditions to the plant biological clock (Kiba et al., 2007). UBP12 and UBP13 are recruited by ZTL and its interacting protein, GIGANTEA (GI), to the ZTL–photoreceptor complex, thereby participating in the regulation of clock cycles by opposing ZTL ubiquitination (Lee et al., 2019).

UBP15 has been linked to the regulation of flowering time and flower size. The *ubp15* mutant displays early flowering and smaller flowers with disrupted regulation of many cell-cycle and flowering genes, but the specific mechanism involved is unknown (Liu et al., 2008). In addition, the SiUBP16 protein is downregulated during phytoplasma infection in the early stages of flower development, suggesting that UBP16 may function in the regulation of floral development in *Sesamum indicum* (Pamei and Makandar, 2022).

#### 5.4.2 Seed development and size control: UBP12/13/14/15/19/26, OsUBP15

During sexual reproduction, the main components of the seed, the embryo and endosperm, are generated through the fusion of the two sperm cells with the egg and the central cell, respectively, to effect double fertilization (Schmidt et al., 2015; Radoeva et al., 2019). Many UBP members play roles in embryo and/or seed development.

Fertilization initiates endosperm and embryo development in flowering plants. However, several UBP mutants exhibit autonomous endosperm development without fertilization. For example, UBP12 and UBP13, which physically interact with LIKE HETEROCHROMATIN PROTEIN 1 (LHP1), are required for the repression of many PcG target genes, such as those involved in seed development. Loss-of-function of *UBP12* and *UBP13* induces fertilization-independent endosperm development (Derkacheva et al., 2016). The *ubp26* mutant exhibits an autonomous endosperm phenotype in the absence of fertilization and arrested seed development after self-pollination (Luo et al., 2008). The PcG target gene *PHERES 1* (*PHE1*) is upregulated, and the level of H3K27me3 at the *PHE1* locus is decreased in the *ubp26* mutant (Luo et al., 2008). Moreover, the *ubp26* mutation induces abnormalities in seed development, including arrested embryo development, shorter siliques, and shriveled seeds (Luo et al., 2008). Interestingly, although UBP26 and UBP12/13 are not members of the same group, the early abnormal seed development phenotype in their mutants is very similar, indicating that the mechanisms by which these UBPs regulate the endosperm phenotype in seed development may be complex.

Seed size, as an important agronomic trait, is determined by the coordinated growth of the embryo, endosperm, and integument (seed coat). UBP15 is widely recognized to be a crucial gene involved in the regulation of seed size (Chen et al., 2024). In *Arabidopsis*, UBP15 acts downstream of DA1 and positively regulates cell proliferation in the outer integument, which determines the seed size. In the *ubp15* mutant, a small seed phenotype is caused by repression of cell proliferation in the outer integument (Du et al., 2014). In rice, OsUBP15 shows a similar function. *OsUBP15* overexpression lines show enhanced grain width and size, whereas *Osubp15* mutants have narrower and smaller grains (Shi et al., 2019), revealing a highly conserved role of UBP15 in seed-size control. The receptor-like kinase ERECTA (ER) is involved in seed-size regulation via the MAPK–DA1–UBP15 pathway (Wu et al., 2022). Specifically, ER-activated MPK3/6 directly interacts with and phosphorylates DA1, leading to the destabilization of the DA1 protein, resulting in the accumulation of the UBP15 protein and promoting the proliferation of outer integument cells (Wu et al., 2022).

Embryo developmental arrest is observed in certain *ubp* mutants. For example, *ubp14* mutants (*ubp14* and *ttn6*) display growth arrest at the globular stage of embryogenesis and accumulate abundant ubiquitinated proteins (Doelling et al., 2001). A similar arrest of embryogenesis has been observed following T-DNA mutation of *UBP19* (Liu et al., 2008).

**Figure 6:**
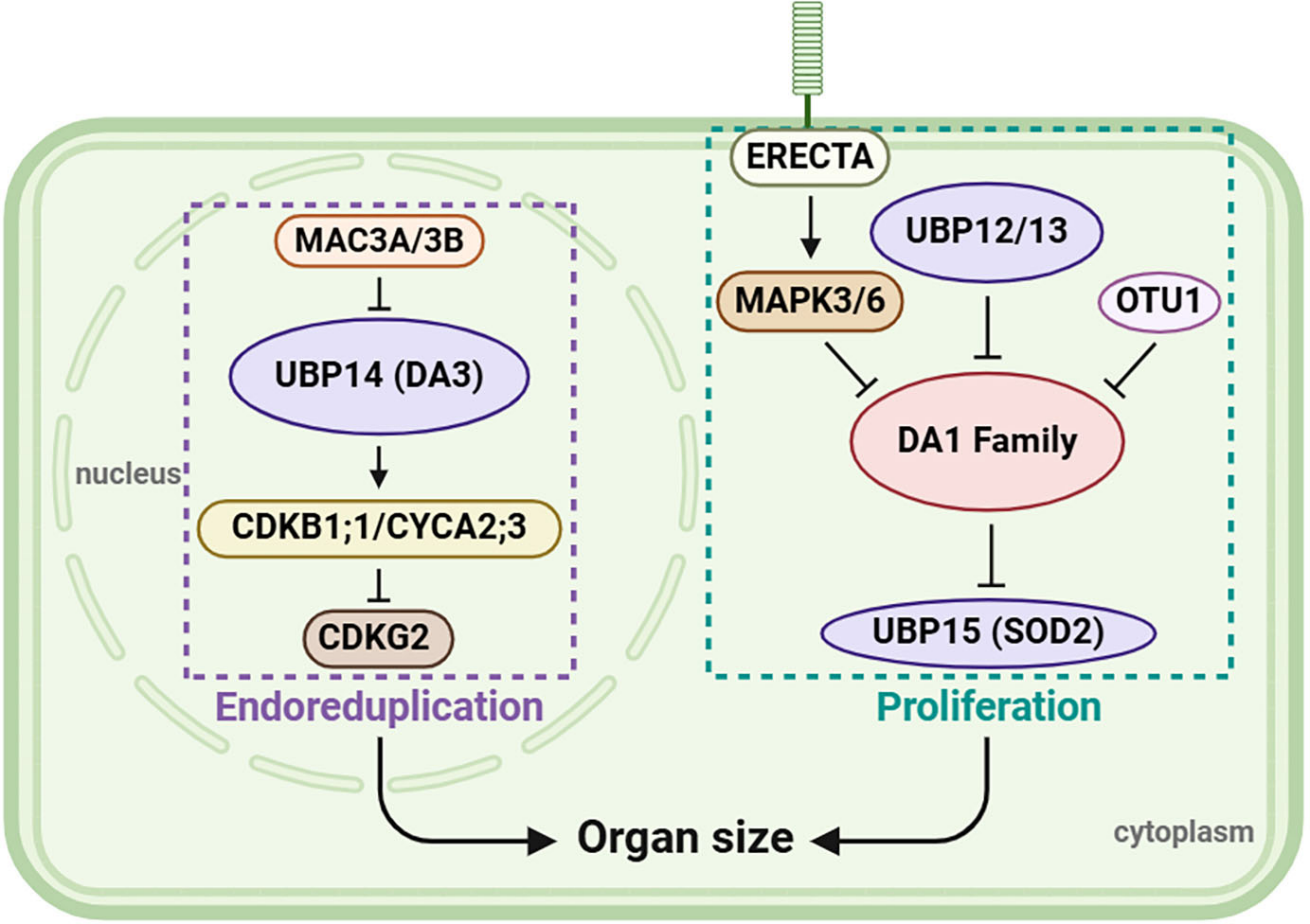
A possible model of organ size regulation by UBPs. UBP12, UBP13, UBP14, and UBP15 together play a role in regulating organ size. On the left of the figure, MAC3A and MAC3B function redundantly in regulating UBP14/DA3 stability, and UBP14 maintains the abundance of CDKB1;1 and CYCA2;3 which then suppress CDKG2 to control endoreduplication. On the right, ER-mediated signaling via MAPK–DA1–UBP15 acts with UBP12, UBP13, and OTU1 to regulate cell proliferation. This figure was created on the BioRender website (https://app.biorender.com/).

The effect of UBPs on the growth and development of flowers, leaves, and seeds can be broadly summarized as serving a regulatory role in organ development, a function of considerable importance for biomass or yield production. According to current knowledge, the regulatory patterns of UBPs are summarized in **Figure 6**, revealing that UBP genes play a crucial role in organ growth and size control. In addition, a deubiquitinating enzyme, the otubain-like cysteine protease OTU1, acts as a transcriptional repressor of *DA1* and *DA2* to regulate seed and organ size (Keren et al., 2020), suggesting that a more intricate regulatory network, reliant on deubiquitinase activity, may operate. Meanwhile, studies have shown that UBP12/13/15 are localized both in the cytoplasm and nucleus (Liu et al., 2008; Cui et al., 2013); thus, more analysis is required in the region where signals are produced and conveyed in the future.

## 6 Morphogenesis

### 6.1 Mitochondrial morphogenesis: UBP27

Two large GTPases, DYNAMIN-RELATED PROTEIN 3A (DRP3A) and DRP3B, are considered to function as primary mitochondrial fission factors and participate in mitochondrial morphogenesis (Arimura and Tsutsumi, 2002; Yoshinaga et al., 2006; Aung and Hu, 2012). UBP27 is an outer mitochondrial membrane protein, in the N_in_–C_out_ orientation, that may participate in mitochondrial morphogenesis by regulating organelle division protein activity (Pan et al., 2014). The association between mitochondria and DRP3 is reduced in *UBP27* overexpression lines, resulting in the alteration of mitochondrial morphology from a rod to a spherical shape (Pan et al., 2014).

### 6.2 Photomorphogenesis: UBP12/13/22

Photomorphogenesis is a process in which plants rely on light to control cell differentiation and structural and functional changes and ultimately form tissues and organs, interconnecting with the shade-avoidance response (SAR), which is an adaptive morphological and physiological change in response to competition for light. PHYTOCHROME INTERACTING FACTORS (PIFs) are crucial regulators of the transition from skotomorphogenesis to photomorphogenesis, exerting a negative regulatory effect by inhibiting photomorphogenesis and maintaining the skotomorphogenic state of etiolated seedlings in the dark (Leivar et al., 2011; Pham et al., 2018). In *Arabidopsis*, the catalytic activity of UBP12/13 is necessary for SAR via deubiquitination and stabilization of PIF7, and the *ubp12* and *ubp13* mutants display impaired sensitivity to shade (Zhou et al., 2021). Under blue light, UBP12 and UBP13 negatively regulate hypocotyl development mediated by the CRYPTOCHROME 2 (CRY2) blue-light receptor (Lindback et al., 2022). UBP12/13 interact with the CRY2/COP1 complex, promoting stabilization of the COP1 ubiquitin ligase and CRY2 ubiquitination (Lindback et al., 2022).

*DE-ETIOLATED 1* (*DET1*) encodes a photoresponsive protein that operates downstream of multiple photoreceptors in plants, and regulates morphogenesis and gene expression by opposing the deubiquitination activity of a deubiquitination module (DUBm), which comprises SAGA-associated factor 11 (SGF11), ENY2, and UBP22 (Nassrallah et al., 2018). UBP22 is a yeast Ubp8 homolog cooperating with SGF11 and ENY2 in H2Bub deubiquitination, while the DET1- and DDB1-Associated 1 (DDA1) protein targets SGF11 for degradation, linking the DET1 complex to light-dependent ubiquitin-mediated proteolytic degradation of the DUBm (Nassrallah et al., 2018). It is noteworthy that UBP26 is also involved in the deubiquitination of H2Bub and influences the expression of flowering regulatory genes, such as *FLC* (Schmitz et al., 2009), but its pathway appears to be distinct from that of UBP22.

## 7 Stress response regulated by UBPs

Plant stress refers to the state in which plants grow under non-optimal conditions; it may lead to deficient growth, reduced crop yield, permanent damage, or death if the stress exceeds plant tolerance (Zhang et al., 2022; Du et al., 2024). Plant stress factors are categorized into two main types: abiotic and biotic. Abiotic factors encompass the various environmental factors that affect plant growth (such as light, water, and temperature), whereas biotic factors encompass other organisms that share the environment with, and interact with, a plant (such as pathogens and pests) (Zhu, 2016). Recent research has illuminated the multifaceted roles of UBPs in plant stress response (**Table 1**) (Zhou et al., 2017; Wu et al., 2019b; Luo et al., 2023).

### 7.1 Salt tolerance: UBP16, ZmUBP15/16/19

The primary environmental factor impeding plant development and productivity is salinity, which affects protein synthesis, photosynthesis, energy, and lipid metabolism (Allakhverdiev et al., 2000; Parida and Das, 2005; Liu et al., 2011). Serine hydroxymethyltransferase 1 (SHM1) is involved in the photorespiratory pathway and influences the capacity to resist biotic and abiotic stress factors (Moreno et al., 2005). UBP16 positively regulates plasma membrane Na^+^/H^+^ antiport activity under salt stress by modifying sodium transport and stabilizing SHM1 (Zhou et al., 2012). Specifically, the *ubp16* mutant exhibits hypersensitivity to saline stress, accumulating more sodium and less potassium than the wild type (Zhou et al., 2012). Moreover, *ubp16* mutant plants show reduced root growth under cadmium exposure, demonstrating that UBP16 also responds to heavy metal stress (Zhao et al., 2013). In maize, overexpression of *ZmUBP15* or *ZmUBP16* rescues the defective phenotype of *ubp16–1* under cadmium stress and partially rescues the salt-stress phenotype of *ubp16–1* (Kong et al., 2019). In the UBP subfamily, UBP15/16/17/18/19 belong to the same group. These results suggest that members of this group may play a common role in the regulation of the cadmium/salt stress response, comparable to the functional redundancy of UBP15/16/17 in the regulation of organ growth.

### 7.2 Inorganic phosphate deficiency: UBP14

Low inorganic phosphate (Pi) availability is frequently the primary limiting factor for plant growth and development. To counteract Pi limitation, plants have evolved sophisticated adaptive responses (Sanchez-Calderon et al., 2005; Peret et al., 2011). For instance, plants inhibit taproot growth, increase lateral root formation, and produce root hairs to adapt their root system to Pi deficiency (Peret et al., 2011). UBP14 is involved in root responses to Pi deficiency in *Arabidopsis*. The *phosphate deficiency root hair defective 1* (*per1*) mutant, identified as a *ubp14* allele from among ethyl methane sulfonate-mutagenized lines, fails to respond to Pi starvation by increasing root hair length and frequency (Li et al., 2010), indicating that UBP14 is crucial for adapting root development to local phosphorus availability. Notably, the *per1* mutation leads to a synonymous substitution in the 12th exon, seemingly initiating a causative lower abundance of UBP proteins, and transcriptional profiling of *per1* plants subjected to Pi starvation revealed genes that are involved in the Pi-deficiency response (Li et al., 2010). However, to elucidate the function of UBP14 in Pi-deficiency signaling pathways, additional research is warranted.

### 7.3 Jasmonic acid signaling: UBP12/13

Jasmonic acid (JA) regulates plant growth and development and plays a crucial role in the response to external injury and pathogen infection (Wang et al., 2021). MYC2 is a bHLH transcription factor that occupies a central position within the JA signaling pathway, participating in regulating the JA-induced response in plants (Kazan and Manners, 2013; Zhu et al., 2023). UBP12 and UBP13 interact with MYC2 and deubiquitinate its protein by removing K63-linked polyubiquitin chains, thereby serving as positive regulators in JA-mediated responses (Jeong et al., 2017).

### 7.4 Brassinosteroid signaling: UBP12/13

Internalization, recycling, and degradation by ubiquitin serve as crucial mechanisms for regulating the activity and abundance of plasma membrane-localized proteins. UBP12 and UBP13 target the plasma membrane-localized brassinosteroid (BR) receptor BR INSENSITIVE 1 (BRI1) and maintain BRI1 stability through deubiquitination (Luo et al., 2022). The *ubp12 ubp13* double mutant exhibits reduced sensitivity to BRs and severe growth defects with decreased abundance of the BRI1 protein, leading to negative modulation of its vacuolar targeting and degradation (Luo et al., 2022). BRI1-EMS-SUPPRESSOR 1 (BES1), a core member of the BR signaling pathway, is deubiquitinated and stabilized by UBP12 and UBP13 (Park et al., 2022; Xiong et al., 2022). In addition, *UBP12/13* expression is induced during recovery after carbon starvation, leading to accumulation and rapid recovery of BES1 in stressed plants (Xiong et al., 2022). Interestingly, a role in nutrient stress response is also indicated in the interaction between UBP12/13 and the E3 ligase ATL3 (Luo et al., 2022), suggesting that UBP12/13 may serve important and broad-ranging functions under nutrient stress.

### 7.5 Auxin signaling: UBP14

Auxins regulate the growth rate of stems, inhibit lateral bud development, and promote rooting. UBP14/TARANI (TNI) is necessary for the optimal auxin response (Majumdar et al., 2020). The *tni* mutation is a hypomorphic allele of *UBP14*, conferring a reduced auxin response and numerous auxin-related phenotypic defects, including an aberrant early cell division pattern during embryogenesis, tricotyledonous and rootless seedlings, irregular root gravitropism, and decreased lateral root formation (Majumdar et al., 2020). Molecular studies have revealed that inefficient splicing of UBP14/TNI transcripts in the *tni* mutant results in the formation of an inactive UBP14 protein, leading to the accumulation of polyubiquitin chains and an excess of polyubiquitinated proteins associated with the auxin pathway (Majumdar et al., 2020).

### 7.6 Abscisic acid signaling: UBP12/13/24, NbUBP12

Abscisic acid (ABA) acts as a signal molecule to mediate tolerance to salinity and drought, specifically by influencing stomatal closure to resist water shortage or maintain cell salt homeostasis (Lopez-Molina et al., 2001; Finkelstein et al., 2002). Ubiquitination plays a crucial role in the ABA signaling pathway (Zhang et al., 2005; Liu et al., 2011; Irigoyen et al., 2014; Baek et al., 2019). UBP24 and UBP12/13 have been identified as DUBs that play a role in this pathway. Sensitivity to ABA increases in young seedlings but decreases in guard cells of the *ubp24* mutant, implying that UBP24 is a negative regulator of ABA signaling (Zhao et al., 2016). In *Nicotiana benthamiana*, NbUBP12 positively regulates drought resistance, which is associated with ABA-mediated stomatal regulation (Lim et al., 2021a). UBP12 and UBP13 regulate drought tolerance and ABA signaling by stabilizing and deubiquitinating the Endosomal sorting complex required for transport I (ESCRT-I) component VACUOLAR PROTEIN SORTING 23A (VPS23A), which recognizes ABA receptors for endosomal degradation (Yu et al., 2016; Liu et al., 2022). In addition to directly regulating VPS23A, UBP12 and UBP13 are involved in stabilizing the E3 ligase XB3 homolog 5 (XBAT35.2) under ABA treatment, which is involved in the ubiquitination of VPS23A, revealing the molecular mechanism by which UBP12 and UBP13 fine-tune the ubiquitination of VPS23A (Liu et al., 2022). Furthermore, research on pepper indicates that CaUBP12 (a homolog of AtUBP12/13) contributes to ABA signaling and the dehydration stress response by inhibiting the degradation of the Ser/Thr protein kinase CaSnRK2.6 (Lim et al., 2021b).

### 7.7 DNA damage response: UBP12/13

DNA damage commonly occurs in living organisms and can arise endogenously or triggered by external genotoxins, such as ionizing radiation, ultraviolet light, and chemical mutagens (Britt, 1996; Manova and Gruszka, 2015). Such damage can disrupt cellular function and impair plant growth and development (Tuteja et al., 2001; Bray and West, 2005). UBP12 and UBP13 are involved in ultraviolet radiation tolerance, possibly in a similar manner to the homolog USP7 in humans (Al Khateeb et al., 2019). UBP12/13 oppose the roles of CRY in DNA damage response (Hu et al., 2023). CRYs, which evolved from bacterial photolyases required for the repair of damaged DNA, enable plants to detect blue light and fine-tune growth and development (Chaves et al., 2011). UBP13 interacts with CRY2 (Hu et al., 2023), but whether UBP13 deubiquitinates CRY2 has not been studied.

### 7.8 Canavanine resistance: UBP1/2/6

Canavanine (CAN) is a non-protein antiherbivore compound produced in certain legumes and can substantially alter the structure and charge of target proteins (Bell, 1960; Rosenthal, 1977, 1986). The toxicity of CAN depends on whether it can replace Arg during translation (Rosenthal and Dahlman, 1986). Compared with wild-type plants, the *ubp1* and *ubp2* single mutants show no phenotypic abnormalities under normal or stress conditions (Yan et al., 2000). However, the *ubp1 ubp2* double mutant exhibits growth abnormalities when treated with CAN, including chlorotic leaves, shorter roots, and reduced fresh weight and growth rate (Yan et al., 2000), suggesting that UBP1 and UBP2 redundantly act to resist CAN. In addition, the CAN resistance of yeast *ubp6* mutants can be restored by UBP6, which interacts with calmodulin (CAM) (Moon et al., 2005).

### 7.9 Immune response against pathogens: UBP2/12/13, OsUBP2, NtUBP12

Salicylic acid (SA), generally produced after pathogen infection, plays a crucial role in plant immunity. NON-EXPRESSER OF PATHOGENESIS-RELATED GENES (NPRs) can bind to SA and mediate SA signaling (Chen et al., 2019). UBP12 and UBP13 interact with NPR3 in an SA-dependent manner and deubiquitinate NPR3 to prevent its degradation and suppress plant immunity that is partially dependent on NPR3/NPR4 functions (Zhou et al., 2023). In response to *Pseudomonas syringae* pv. *tomato* (*Pst* DC3000) inoculation, UBP12/13 and their homolog NtUBP12 in tobacco function as negative regulators of Cf-9-dependent hypersensitivity (Ewan et al., 2011). In addition, UBPs participate in the response to reactive oxygen species, which act as signaling molecules for diverse stimuli. In rice, *Osubp2* exhibits enhanced resistance to bacterial blight and leaf blast caused by *Xanthomonas oryzae* pv. *oryzae* and *Magnaporthe oryzae*, respectively, whereas *OsUBP2* overexpression causes chloroplast structural defects, programmed cell death, and reactive oxygen species accumulation, resulting in reduced resistance to rice blast (Jiang et al., 2022a).

## 8 Conclusion

In plants, ubiquitination has been linked to numerous cellular processes, serving as a crucial signaling mechanism for plant development, morphogenesis, and stress response (Moon et al., 2004; Bartel and Citovsky, 2012; Sadanandom et al., 2012). Protein ubiquitination requires the sequential activities of E1 ubiquitin-activating enzymes, E2 ubiquitin-conjugating enzymes, and E3 ubiquitin ligases (Pickart, 2001; Pickart and Eddins, 2004). Seven lysine residues in ubiquitin, together with methionine at its N-terminus, are capable of self-modification, facilitating the formation of polyubiquitin chains with different types of linkages (Pickart and Eddins, 2004; de Poot et al., 2017). Different chain types are involved in various cellular processes and play unique signaling roles. A large body of evidence indicates that not only ubiquitinating enzymes but also deubiquitinating enzymes/deubiquitinases (DUBs) can regulate the fate of substrates in response to plant development, morphogenesis, and stress response, and research into this is increasing (Luo et al., 2023). Sullivan et al. (1990) first detected deubiquitinating activity in wheat germ in 1990. After almost 10 years, the *Arabidopsis* genome was sequenced, and UBPs, the largest family of DUBs, were identified (Yan et al., 2000). Early reports of UBPs in plants primarily focused on structure and biochemical activities, whereas more recent reports have employed advanced technologies to systematically explore the biological functions of UBPs. In this review, we have highlighted the roles of UBPs in plant growth and development, morphogenesis, and stress response and briefly reviewed the known identification of UBPs and their interacting proteins, which is beneficial for understanding UBP-mediated biological mechanisms in plants.

Current research on UBP members has demonstrated their diverse functions in regulating plant organ size and development (Du et al., 2014; Derkacheva et al., 2016; Cao et al., 2022), sexual reproduction (Doelling et al., 2007), photomorphogenesis (Lindback et al., 2022), hormone signaling (Zhao et al., 2016; Xiong et al., 2022), and immunological response (Jiang et al., 2022a; Zhou et al., 2023). The same UBP can perform multiple functions, typically exemplified by the UBP12/13 group. While initially identified as negative regulators of plant immunity (Ewan et al., 2011), UBP12/13 subsequently emerged as critical players in the control of photoperiodic flowering by regulating clock gene expression (Cui et al., 2013). UBPs are involved in many plant hormone signaling pathways, including JA, BR, and ABA responses (Jeong et al., 2017; Liu et al., 2022; Xiong et al., 2022). Recent studies have further revealed their roles in the regulation of DNA damage repair (Hu et al., 2023) and ultraviolet radiation tolerance (Al Khateeb et al., 2019). In addition, the same function can be modulated by different UBPs. For instance, deubiquitination of H2A can be mediated by UBP12 and UBP13 (Kralemann et al., 2020). UBP5 acts as a primary histone H2A deubiquitinase, which is a component of the large version of the PEAT complex and is involved in transcriptional and development regulation at the whole-genome level in plants (Zheng et al., 2023). As **Figure 6** illustrates, UBP12 and UBP13 can act upstream of the DA1 family, while UBP15 functions downstream, implying complex roles of different UBP family members in the regulation of plant organ size. Compared with the versatile functions of UBP12/13, we know very little about the functions of other UBPs, and thus, there is considerable scope for further functional studies of UBPs. Although some interaction partners have been identified, the identification of UBP enzyme substrates is a critical breakthrough in research on UBP mechanisms, but progress in this area has been limited, possibly because of the weak binding of UBPs with their substrate and the short binding time.

UBPs constitute the largest group within the plant DUB family. Research into their functions has considerably advanced our understanding of the physiological roles of ubiquitin-mediated modifications in plants. In contrast to the vast array of E3 enzymes that oppose UBP function, UBPs exhibit a diversity and complexity of functions, offering valuable insights into the molecular regulatory mechanisms in plants. As enzymes, substrate identification of UBPs remains the most critical aspect for elucidating their mechanism. Despite a broad understanding of their molecular functions, the substrates of the vast majority of UBPs remain unknown. The sluggish progress in this area is likely due to the dynamic nature of the regulation of ubiquitination levels, which results in the transient and weak association between deubiquitinating enzymes and their substrates, making it challenging to identify substrates using traditional methods. Recently, the emergence of novel protein interaction detection technologies holds promise for the identification of UBP substrates and the analysis of UBP functions.

## Author contributions

XW: Writing – original draft, Methodology, Visualization. XL: Writing – review & editing, Conceptualization, Data curation. KS: Writing – review & editing. LD: Writing – review & editing, Conceptualization, Supervision.
